# On Fuzzy Congruences and Fuzzy Strong *h*-Ideals of Hemirings

**DOI:** 10.1155/2014/975474

**Published:** 2014-07-17

**Authors:** Kuanyun Zhu, Jianming Zhan, Yunqiang Yin

**Affiliations:** ^1^Department of Mathematics, Hubei Minzu University, Enshi, Hubei 445000, China; ^2^Faculty of Science, Kunming University of Science and Technology, Kunming, Yunnan 650093, China

## Abstract

The aim of this paper is to introduce the concepts of fuzzy strong *h*-ideals and fuzzy congruences of hemirings. The quotient hemirings via fuzzy strong *h*-ideals are investigated. The relationships between fuzzy congruences and fuzzy strong *h*-ideals of hemirings are discussed. Pay attention to an open question on fuzzy congruences. Finally, the normal fuzzy strong *h*-ideals of hemirings are explored.

## 1. Introduction

The concept of fuzzy sets was formulated by Zadeh [1], and since then there has been a remarkable growth of fuzzy set theory. In 1989, Filep and Maurer [2] introduced the concepts of fuzzy congruences and compatible partitions. Further, Zadeh [3], Murali [4], and Kuroki [5] discussed the properties of fuzzy congruences. Moreover, Dutta and Biswas [6] redefined fuzzy equivalent relations and fuzzy congruences of semirings.

Semirings, regarded as a generalization of rings, have been recently found particularly useful in solving problems in different disciplines of applied mathematics and information sciences because semirings provide an algebraic framework for modelling. A special semiring with a zero and endowed with the commutative addition is said to be a hemiring. Nowadays, semirings (hemirings) are useful in optimization theory, graph theory of discrete event dynamical systems, matrices, determinants, generalized fuzzy computation, automata, theory, formal language theory, coding theory, and analysis of computer programs.

We know that ideal theory of semirings plays a central role in the structure theory and is useful for many purposes. The properties of *h*-ideals and *k*-ideals of hemirings were thoroughly investigated by la Torre [7]. Further, fuzzy *k*-ideals of semirings were investigated by [8–12]. In 2004, Jun et al. [13] introduced the concept of fuzzy *h*-ideals of hemirings and investigated some related properties of hemirings. In particular, the *h*-hemiregular hemirings were described by Zhan and Dudek [14]. Now, many researchers investigated hemirings, such as Ma et al. [15–20].

In this paper, we study fuzzy congruences and fuzzy strong *h*-ideals of hemirings. The remaining part of this paper is organized as follows. In Section 2, we first recall some basic definitions of hemrings and give the concepts of strong *h*-ideals and fuzzy strong *h*-ideals of hemirings. In Section 3, we consider fuzzy homomorphisms of hemirings and the quotient hemirings via fuzzy strong *h*-ideals. In Section 4, we investigate the relationships between fuzzy congruences and fuzzy strong *h*-ideals. In Section 5, we introduce normal fuzzy strong *h*-ideals of hemirings.

## 2. Preliminaries

Recall that a semiring is an algebraic system (*S*, +, ·) consisting a nonempty set of *S* together with two binary operations on *S* called an addition and a multiplication (denoted in the usual manner) such that (*S*, +) and (*S*, ·) are semigroups and the following distributive laws: *a*(*b* + *c*) = *ab* + *ac* and (*a* + *b*)*c* = *ac* + *bc* are satisfied for all *a*, *b*, *c* ∈ *S*.

By zero of a semiring (*S*, +, ·) we mean an element 0 ∈ *S* such that 0 · *x* = *x* · 0 = 0 and 0 + *x* = *x* + 0 = *x* for all *x* ∈ *S*. By an identity of a semiring (*S*, +, ·) we mean an element 1 ∈ *S* such that 1 · *x* = *x* · 1 = 1 for all *x* ∈ *S*. A semiring with a zero and a commutative semiring (*S*, +) is called a hemiring. Throughout this paper, *S* is always a hemiring.

A subset *A* of *S* is called a left(right) ideal of *S* if *A* is closed under addition and *SA*⊆*A*(*AS*⊆*A*). A left ideal *A* of *S* is called a left *h*-ideal if for any *x*, *z* ∈ *S*, and *a*, *b* ∈ *A* and *x* + *a* + *z* = *b* + *z*, it follows *x* ∈ *A*. A right *h*-ideal is defined analogously. A left ideal *A* of *S* is called a strong left *h*-ideal if for any *x*, *y*, *z* ∈ *S* and *a*, *b* ∈ *A* from *x* + *a* + *z* = *y* + *b* + *z*, it implies *x* ∈ *y* + *A*. A strong right *h*-ideal is defined analogously. Every strong *h*-ideal is an *h*-ideal; the converse is not true [20].

A fuzzy set *μ* of *S* is defined as a mapping from *μ* to the real interval [0, 1]. Let *F*(*S*) denote the set of all fuzzy sets of *S*. A fuzzy set *μ* in *S* of the form

(1) $$\begin{array}{c} \mu \left(x\right)=\{\begin{array}{cc} r & \text{if}\;\;x=y,\\ 0 & \text{if}\;\;x\neq y, \end{array} \end{array}$$

is called a fuzzy point with support *y* and value *r* and is denoted by *y*_*r*_. In particular, if *r* = 1, *y*_1_ denotes the fuzzy point with support *y* and value 1.

**Definition 1 (see [13]).**

A fuzzy set *μ* of *S* is called a fuzzy left ideal if for all *x*, *y* ∈ *S*, we have

(F_1_) *μ*(*x* + *y*) ≥ *μ*(*x*)∧*μ*(*y*),
(F_2_) *μ*(*xy*) ≥ *μ*(*y*).

A fuzzy left ideal *μ* of *S* is called a fuzzy left *h*-ideal if for all *a*, *b*, *x*, *z* ∈ *S*, *x* + *a* + *z* = *b* + *z* → *μ*(*x*) ≥ *μ*(*a*)∧*μ*(*b*). A fuzzy right *h*-ideal is defined similarly.

**Definition 2 (see [20]).**

Let *μ* and *ν* be fuzzy sets of *S*. The sum *μ* + *ν* of *μ* and *ν* is defined by

(2) $$\begin{array}{c} \left(\mu +\nu \right)\left(x\right)=\underset{x=a+b}{⋁}\mu \left(a\right)\wedge \nu \left(b\right). \end{array}$$

In particular,

(3) $$\begin{array}{c} \left(y+\mu \right)\left(x\right)=\underset{x=y+a}{⋁}\mu \left(a\right). \end{array}$$

Note that if *x* ∈ *S* and *μ* ∈ *F*(*S*), then *y* + *μ* = *y*_1_ + *μ*.

**Definition 3 .**

A fuzzy set *μ* of *S* is called a fuzzy strong left(right) *h*-ideal of *S* if for all *x*, *y*, *z*, *a*, *b* ∈ *S*,

1. *μ*(*x* + *y*) ≥ *μ*(*x*)∧*μ*(*y*),
2. *μ*(*xy*) ≥ *μ*(*y*)(*μ*(*xy*) ≥ *μ*(*x*)),
3. *x* + *a* + *z* = *y* + *b* + *z* → (*y*_1_ + *μ*)(*x*) ≥ *μ*(*a*)∧*μ*(*b*).

Note that if *μ* is a fuzzy strong *h*-ideal of *S*, then *μ*(0) ≥ *μ*(*x*).

**Example 4 .**

Let *S* = {0, *a*, *b*, *c*} be a set with an addition operation (+) and a multiplication operation (·) as follows:

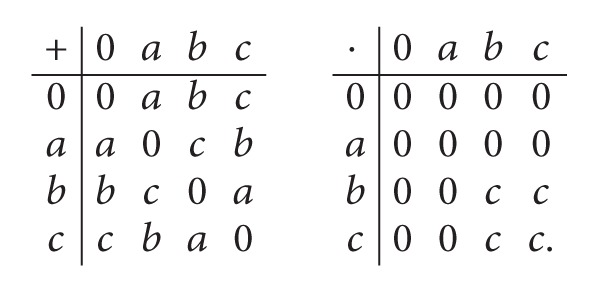
(4)

Define a fuzzy set *μ* in *S* by *μ*(0) = *μ*(*a*) = 0.6, *μ*(*b*) = *μ*(*c*) = 0.5. Then *μ* is a fuzzy strong *h*-ideal of *S*.

**Definition 5 (see [18]).**

Let *μ* and *ν* be fuzzy sets of *S*. Then *h*-sum of *μ* and *ν* is defined by

(5) (μ+hν)(x)=⋁x+a′+b′+z=a′′+b′′+z(μ(a′)∧μ(a′′)∧ν(b′)∧ν(b′′)),

if there exist *a*′, *a*′′, *b*′, *b*′′, *z* ∈ *S* such that *x* + *a*′ + *b*′ + *z* = *a*′′ + *b*′′ + *z*; otherwise, (*μ*+_*h*_ *ν*)(*x*) = 0.

Let *μ* be a fuzzy set of *S* and *r* ∈ [0, 1]. Then the sets *μ*_*r*_ = {*x*∣*μ*(*x*) ≥ *r*} and *μ*_*r*_^*s*^ = {*x*∣*μ*(*x*) > *r*} are called an *r*-level subset and an *r*-strong level of *μ*, respectively. We now characterize the fuzzy strong *h*-ideal of *S* by using their (strong) level subsets.

**Theorem 6 .**

A fuzzy set *μ* of *S* is a fuzzy strong *h*-ideal of *S* if and only if nonempty *μ*_*r*_ is a strong *h*-ideal of *S* for all *r* ∈ [0, 1].

**Proof**

Let *μ* be a fuzzy strong *h*-ideal of *S*, *x*, *y* ∈ *μ*_*r*_, and *a* ∈ *S*. Then *μ*(*x* + *y*) ≥ *μ*(*x*)∧*μ*(*y*) ≥ *r*, *μ*(*ax*) ≥ *μ*(*a*)∨*μ*(*x*) ≥ *r*, and so *x* + *y*, *ax* ∈ *μ*_*r*_. Similarly, we get *xa* ∈ *μ*_*r*_, hence *μ*_*r*_ is an ideal of *S*.

Now, let *x*, *y*, *z* ∈ *S* and *a*, *b* ∈ *μ*_*r*_ be such that *x* + *a* + *z* = *y* + *b* + *z*. Hence, *μ*(*a*) ≥ *r*, *μ*(*b*) ≥ *r*. Thus we have

(6) (y1+μ)(x)=⋁x=y+b′μ(b′)≥μ(a)∧μ(b)≥r.

This implies that there exists *b*′ ∈ *S* such that *x* = *y* + *b*′ and *μ*(*b*′) ≥ *r*; that is, *b*′ ∈ *μ*_*r*_, and so *x* ∈ *y* + *μ*_*r*_. Therefore, *μ*_*r*_ is a strong *h*-ideal of *S*.

Conversely, assume that the given conditions hold. Let *x*′, *y*′ ∈ *S*. If possible, let *μ*(*x*′ + *y*′) < *μ*(*x*′) ∧ *μ*(*y*′). Choose *r* such that *μ*(*x*′ + *y*′) < *r* < *μ*(*x*′) ∧ *μ*(*y*′). Then *x*′, *y*′ ∈ *μ*_*r*_, but *x*′ + *y*′ ∉ *μ*_*r*_, a contradiction. Hence, *μ*(*x* + *y*) ≥ *μ*(*x*)∧*μ*(*y*) for all *x*, *y* ∈ *S*. Similarly, we have *μ*(*xy*) ≥ *μ*(*x*)∨*μ*(*y*) for all *x*, *y* ∈ *S*.

Now assume there exist *x*′, *y*′, *z*′, *a*′, *b*′ ∈ *S* such that *x*′ + *a*′ + *z*′ = *y*′ + *b*′ + *z*′ and (*y*_1_′ + *μ*)(*x*′) < *μ*(*a*′)∧*μ*(*b*′); choose *r* such that (*y*_1_′ + *μ*)(*x*′) < *r* < *μ*(*a*′)∧*μ*(*b*′). Then *a*′, *b*′ ∈ *μ*_*r*_, but (*y*_1_′ + *μ*)(*x*′) = ⋁_*x*′=*y*′+*c*_ *μ*(*c*) < *r*. That is, *x*′ ∉ *y*′ + *μ*_*r*_, a contradiction. Hence, (*y*_1_ + *μ*)(*x*) ≥ *μ*(*a*)∧*μ*(*b*) for all *x*, *y*, *z*, *a*, *b* ∈ *S* with *x* + *a* + *z* = *y* + *b* + *z*. This implies that *μ* is a fuzzy strong *h*-ideal of *S*.

## 3. Quotient Hemirings and Their Isomorphisms

In this section, the quotient hemirings via fuzzy strong *h*-ideals are investigated. Finally, we give an isomorphism theorem of hemirings.

Let *θ* be an equivalence relation on *S*. Recall that *θ* is called a congruence relation on *S* if (*a*, *b*) ∈ *θ* and (*c*, *d*) ∈ *θ* imply (*a* + *c*, *b* + *d*) ∈ *θ* and (*ac*, *bd*) ∈ *θ*.

Let *I* be a strong *h*-ideal of *S*, *x*, *y* ∈ *S*. We call *x* congruent to *y* mod *I*, if and only if there exist *a*, *b* ∈ *I*and *z* ∈ *S* be such that *x* + *a* + *z* = *y* + *b* + *z* [20]. It is checked that the relation *x* ≡ *y*(mod⁡  *I*) is a congruence relation on *S*.

**Lemma 7 (see [20]).**

Let *I* be a strong *h*-ideal of *S*. If *x*, *y* ∈ *S*, then

1. *x* ∈ [*y*]_*I*_ if and only if *x* ∈ *y* + *I*,
2. [*x*]_*I*_ + [*y*]_*I*_ = [*x* + *y*]_*I*_,
3. {*ab*∣*a* ∈ [*x*]_*I*_, *b* ∈ [*y*]_*I*_}⊆[*xy*]_*I*_.

Let *μ* be a fuzzy strong *h*-ideal of *S* and *μ*_*r*_ an *r*-level subset of *S*. We denote by *μ*_*x*_ = {*y* ∈ *S*∣*y* ∈ [*x*]_*μ*_*r*__} the equivalence class containing *x* and by *S*/*μ* = {*μ*_*x*_∣*x* ∈ *S*} the set of all equivalence classes of *S*.

**Theorem 8 .**

If *μ* is a fuzzy strong *h*-ideal of *S* and *μ*_*r*_^2^ = *μ*_*r*_, then *S*/*μ* is a hemiring under the binary operations:

(7) $$\begin{array}{c} \mu_{x}+\mu_{y}=\mu_{x+y},\;\;\;\mu_{x}\cdot \mu_{y}=\mu_{xy}, \end{array}$$

for any *x*, *y* ∈ *S*.

**Proof**

Firstly, we show that the above binary operations are well defined. In fact, if *μ*_*x*_ = *μ*_*u*_ and *μ*_*y*_ = *μ*_*v*_. Since *μ*_*x*_ = {*y* ∈ *S*∣*y* ∈ [*x*]_*μ*_*r*__}, we have *μ*_*x*_ = [*x*]_*μ*_*r*__, and so [*x*]_*μ*_*r*__ = [*u*]_*μ*_*r*__, [*y*]_*μ*_*r*__ = [*v*]_*μ*_*r*__. Then [*x*]_*μ*_*r*__ + [*y*]_*μ*_*r*__ = [*u*]_*μ*_*r*__ + [*v*]_*μ*_*r*__. By Lemma 7, we have [*x*]_*μ*_*r*__ + [*y*]_*μ*_*r*__ = [*x* + *y*]_*μ*_*r*__, and so [*x* + *y*]_*μ*_*r*__ = [*u* + *v*]_*μ*_*r*__; that is, *μ*_*x*+*y*_ = *μ*_*u*+*v*_. Hence the addition is well defined.

By Lemma 7, we also know [*x*]_*μ*_*r*__ · [*y*]_*μ*_*r*__⊆[*xy*]_*μ*_*r*__. Next we show [*xy*]_*μ*_*r*__⊆[*x*]_*μ*_*r*__ · [*y*]_*μ*_*r*__. Let *z* = *ab* ∈ [*xy*]_*μ*_*r*__; since *μ*_*r*_^2^ = *μ*_*r*_, then *z* = *ab* ∈ [*xy*]_*μ*_*r*__ = *xy* + *μ*_*r*_ = *xy* + *xμ*_*r*_ + *yμ*_*r*_ + *μ*_*r*_^2^ = (*x* + *μ*_*r*_)(*y* + *μ*_*r*_) = [*x*]_*μ*_*r*__ · [*y*]_*μ*_*r*__. This implies that *z* = *ab* ∈ [*x*]_*μ*_*r*__ · [*y*]_*μ*_*r*__, and so [*xy*]_*μ*_*r*__⊆[*x*]_*μ*_*r*__ · [*y*]_*μ*_*r*__; that is, [*x*]_*μ*_*r*__ · [*y*]_*μ*_*r*__ = [*xy*]_*μ*_*r*__. This means that *μ*_*x*_ · *μ*_*y*_ = *μ*_*xy*_. Hence the multiplication is well defined. Now it is easy to verify that *S*/*μ* is a hemiring.

**Definition 9 (see [21]).**

Let *f* be a homomorphism from *S* to *S*′, *μ* a fuzzy subset of *S*, and *μ*′ a fuzzy subset of *S*′. Then the image *f*(*μ*) of *μ* and the preimage *f* ^−1^(*μ*) of *μ*′ are both fuzzy sets defined, respectively, as follows:

(8) $$\begin{array}{c} f\left(\mu \right)\left(y\right)=\{\begin{array}{cc} \underset{x\in f^{-1}(y)}{⋁}\mu \left(x\right) & \text{if}\;\;f^{-1}\left(y\right)\neq \emptyset ,\\ 0 & \text{if}\;\;f^{-1}\left(y\right)=\emptyset , \end{array}\\ f^{-1}\left(\mu^{\prime }\right)\left(x\right)=\mu^{\prime }\left(f\left(x\right)\right), \end{array}$$

for all *x* ∈ *S*.

**Definition 10 .**

Let *f* : *S* → *S*′ be a homomorphism of hemirings. A strong *h*-ideal *I* of *S* is called *f*-compatible, if for all *x*, *y*, *z*, *a*, *b* ∈ *S*, *f*(*x* + *a* + *z*) = *f*(*y* + *b* + *z*) implies *x* ∈ *y* + *I*. A fuzzy strong *h*-ideal *μ* of *S* is called *f*-compatible, if for all *x*, *y*, *z*, *a*, *b* ∈ *S*, *f*(*x* + *a* + *z*) = *f*(*y* + *b* + *z*) implies (*y* + *μ*)(*x*) ≥ *μ*(*a*)∧*μ*(*b*).

**Remark 11 .**

If the above *f* is a monomorphism, then every fuzzy strong *h*-ideal is *f*-compatible.

**Theorem 12 .**

Let *f* : *S* → *S*′ be an epimorphism of hemirings. If *μ* is a *f*-compatible fuzzy strong *h*-ideal of *S*, then *f*(*μ*) is a fuzzy strong *h*-ideal of *S*′.

**Proof**

(1) Let *x*′, *y*′ ∈ *S*′; then

(9) f(μ)(x′+y′)=⋁f(a)=x′+y′μ(a)≥⋁f(a′)=x′,f(a′′)=y′μ(a′+a′′)≥⋁f(a′)=x′,f(a′′)=y′μ(a′)∧μ(a′′)=⋁f(a′)=x′μ(a′)∧⋁f(a′′)=y′μ(a′′)=f(μ)(x′)∧f(μ)(y′).

(2) Let *x*′, *y*′ ∈ *S*′; then

(10) f(μ)(x′y′)=⋁f(a)=x′y′μ(a)≥⋁f(a′)=x′,f(a′′)=y′μ(a′a′′)≥⋁f(a′)=x′,f(a′′)=y′μ(a′)∨μ(a′′)=⋁f(a′)=x′μ(a′′)∨⋁f(a′′)=y′μ(a′′)=f(μ)(x′)∨f(μ)(y′).

(3) Let *x*, *y*, *z*, *a*, *b* ∈ *S* and *x*′, *y*′, *z*′, *a*′, *b*′ ∈ *S* be such that *x*′ + *a*′ + *z*′ = *y*′ + *b*′ + *z*′, *f*(*x*) = *x*′, *f*(*y*) = *y*′, *f*(*z*) = *z*′, *f*(*a*) = *a*′, *f*(*b*) = *b*′; then we have *f*(*x* + *a* + *z*) = *f*(*y* + *b* + *z*). Since *μ* is *f*-compatible, we have (*y*_1_ + *μ*)(*x*) ≥ *μ*(*a*)∧*μ*(*b*); thus

(11) (y1′+f(μ))(x′)=⋁x′=y′+c′f(μ)(c′)=⋁x′=y′+c′⋁f(c)=c′μ(c)≥⋁x=y+cμ(c)=(y1+μ)(x)≥μ(a)∧μ(b),

and so

(12) (y1′+f(μ))(x′)=⋁f(a)=a′⋁f(b)=b′μ(a)∧μ(b)=⋁f(a)=a′μ(a)∧⋁f(b)=b′μ(b)=f(μ)(a′)∧f(μ)(b′).

Therefore, *f*(*μ*) is a fuzzy strong *h*-ideal of *S*′.

Similarly, we can obtain the following result.

**Theorem 13 .**

Let *f*: *S* → *S*′ be a monomorphism of hemirings. If *μ*′ is a fuzzy strong *h*-ideal of *S*′, then *f* ^−1^(*μ*′) is an *f*-compatible fuzzy strong *h*-ideal of *S*.

**Proof**

The proof is similar to Theorem 12.

**Theorem 14 .**

Let *μ* and *ν* be two fuzzy sets of *S*. If they are fuzzy strong left (resp., right) *h*-ideals of *S*, then so are *μ*∩*ν* and *μ*+_*h*_ *ν*.

**Proof**

We first show that *μ*∩*ν* is a fuzzy strong left *h*-ideal of *S*. For any *x*, *y* ∈ *S*,

(13) (μ∩ν)(x+y)=μ(x+y)∧ν(x+y)=(μ(x)∧μ(y))∧(ν(x)∧ν(y))≥(μ(x)∧ν(y))∧(μ(x)∧ν(y))=(μ∩ν)(x)∧(μ∩ν)(y).

Since *μ*(*xy*) ≥ *μ*(*y*) and *ν*(*xy*) ≥ *ν*(*y*), it follows that

(14) (μ∩ν)(xy)=μ(xy)∧ν(xy)≥μ(y)∧ν(y)=(μ∩ν)(y).

Therefore, *μ*∩*ν* is a fuzzy left ideal of *S*.

Let *a*, *b*, *x*, *y*, *z* be such that *x* + *a* + *z* = *y* + *b* + *z*. Then

(15) (y1+μ∩ν)(x)=⋁x=y+c(μ∩ν)(c)=⋁x=y+c(μ(c)∧ν(c))=⋁x=y+cμ(c)∧⋁x=y+cν(c)≥(μ(a)∧μ(b))∧(ν(a)∧ν(b))=(μ(a)∧ν(a))∧(μ(b)∧ν(b))=(μ∩ν)(a)∧(μ∩ν)(b).

Hence *μ*∩*ν* is a fuzzy strong left *h*-ideal of *S*.

Now we show that *μ*+_*h*_ *ν* is a fuzzy strong left *h*-ideal. In fact,

(1) for any *x*, *y* ∈ *S*, we have

(16) (μ+hν)(x+y) =⋁x+y+a′+b′+z=a′′+b′′+zμ(a′)∧μ(a′′)∧ν(b′)∧ν(b′′) ≥⋁x+c′+d′+z′=c′′+d′′+z′μ(c′)∧μ(c′′)∧ν(d′)∧ν(d′′)  ∧⋁y+e′+f′+z′′=e′′+f′′+z′′μ(e′)∧μ(e′′)∧ν(f′)∧ν(f′′) =(μ+hν)(x)∧(μ+hν)(y).

(2) For any *x*, *y* ∈ *S*, we have

(17) (μ+hν)(y) =⋁y+a′+b′+z=a′′+b′′+zμ(a′)∧μ(a′′)∧ν(b′)∧ν(b′′) ≤⋁xy+xa′+xb′+xz=xa′′+xb′′+xzμ(xa′)  ∧μ(xa′′)∧v(xb′)∧ν(xb′′) ≤⋁xy+c′+d′+z′=c′′+d′′+z′μ(c′)  ∧μ(c′′)∧v(d′)∧ν(d′′) =(μ+hν)(xy).

(3) Let *a*, *b*, *x*, *y*, *z*′ be any elements of *S* such that *x* + *a* + *z*′ = *y* + *b* + *z*′. If there exist *c*′, *c*′′, *d*′, *d*′′, *e*′, *e*′′, *f*′, *f*′′, *z*′′, *z*′′′ ∈ *S*, such that

(18) $$\begin{array}{c} a+c^{\prime }+d^{\prime \prime }+z^{\prime \prime }=c^{\prime \prime }+d^{\prime \prime }+z^{\prime \prime },\\ b+e^{\prime }+f^{\prime }+z^{\prime \prime \prime }=e^{\prime \prime }+f^{\prime \prime }+z^{\prime \prime \prime }, \end{array}$$

then we have

(19) $$\begin{array}{c} x+c^{\prime \prime }+d^{\prime \prime }+e^{\prime }+f^{\prime }+z^{\prime \prime \prime \prime }=y+c^{\prime }+d^{\prime }+e^{\prime \prime }+f^{\prime \prime }+z^{\prime \prime \prime \prime }, \end{array}$$

where *z*′′′′ = *z*′ + *z*′′ + *z*′′′ + *a* + *b*, and so

(20) [y1+(μ+hν)](x) =⋁x=y+c(μ+hν)(c) =⋁x=y+c⋁ c+a′+b′+z=a′′+b′′μ(a′)∧μ(a′′)∧ν(b′)∧ν(b′′) ≥⋁c′+d′+e′′+f′′+a′+b′+z=a′′+b′′μ(a′)  ∧μ(a′′)∧ν(b′)∧ν(b′′) ≥μ(c′′+f′)∧μ(c′+d′+e′′+f′′)  ∧ν(e′+d′′)∧ν(c′′+f′+e′+d′′) ≥μ(c′)∧μ(c′′)∧ν(d′)∧ν(d′′)  ∧μ(e′)∧μ(e′′)∧ν(f′)∧ν(f′′).

This gives

(21) [y1+(μ+hν)](x) ≥⋁a+c′+d′+z′′=c′′+d′′+z′′μ(c′)∧μ(c′′)∧ν(d′)∧ν(d′′)  ∧⋁b+e′+f′+z′′′=e′′+f′′+z′′′μ(e′)∧μ(e′′)∧ν(f′)∧ν(f′′) =(μ+hν)(a)∧(μ+hν)(b).

Otherwise, we have (*μ*+_*h*_ *ν*)(*a*) = 0 or (*μ*+_*h*_ *ν*)(*b*) = 0, and so [*y*_1_ + (*μ*+_*h*_ *ν*)](*x*) ≥ 0 = (*μ*+_*h*_ *ν*)(*a*)∧(*μ*+_*h*_ *ν*)(*b*). Summing up the above statements, *μ*+_*h*_ *ν* is a fuzzy strong left *h*-ideal of *S*. The case for fuzzy strong right *h*-ideals can be similarly proved.

Now denote by FSI(*S*) the set of all fuzzy strong *h*-ideals of *S* with the same tip *t*; that is, *μ*(0) = *ν*(0) for all *μ*, *ν* ∈ FSI(*S*). Then we have the following result.

**Theorem 15 .**

The (FSI(*S*), +_*h*_, ∩) is a bounded complete lattice under the relation “⊆”.

**Proof**

Let *μ*, *ν* ∈ FSI(*S*). It follows from Theorem 14 that *μ*∩*ν* ∈ FSI(*S*) and *μ*+_*h*_ *ν* ∈ FSI(*S*). It is clear that *μ*∩*ν* is the greatest lower bound of *μ* and *ν*. We now show that *μ*+_*h*_ *ν* is the least upper bound of *μ* and *ν*, since *μ*(0) = *ν*(0); for any *x* ∈ *S*, we have

(22) (μ+hν)(x) =⋁x+a′+b′+z=a′′+b′′+zμ(a′)∧μ(a′′)∧ν(b′)∧ν(b′′) ≥μ(0)∧μ(x)∧ν(0)∧ν(0) =μ(0)∧μ(x) =μ(x).

Hence, *μ*⊆*μ*+_*h*_ *ν*. Similarly, we have *ν*⊆*μ*+_*h*_ *ν*. Now, let *ω* ∈ FSI(*S*) be such that *μ*, *ν*⊆*ω*; then we have *μ*+_*h*_ *ν*⊆*ω*+_*h*_ *ω*⊆*ω*. Hence, *μ*∨*ν* = *μ*+_*h*_ *ν*. It is clear that replace the {*μ*, *ν*} with arbitrary family of FSI(*S*) and so (FSI(*S*), +_*h*_, ∩) is a complete lattice under the relation “⊆”. *∅* and *χ*_*S*_ are the minimal and the maximal elements in (FSI(*S*), +_*h*_, ∩), respectively. Therefore, (FSI(*S*), +_*h*_, ∩) is a bounded complete lattice.

Finally, we give an isomorphism theorem of hemirings.

**Theorem 16 .**

Let *f*: *S* → *S*′ be an isomorphism of hemirings and *ν* a fuzzy strong *h*-ideal of *S*′. If *ν*_*r*_^2^ = *ν*_*r*_ and *f* ^−1^(*ν*)_*r*_^2^ = *f* ^−1^(*ν*)_*r*_, then

(23) $$\begin{array}{c} S/f^{-1}\;\left(\nu \right)≅S^{\prime }/\nu . \end{array}$$

**Proof**

It follows from Theorems 8 and 13, *S*/*f* ^−1^(*ν*) and *S*′/*ν* are both hemirings. Define *ξ* : *S*/*f* ^−1^(*ν*) → *S*/*ν* by

(24) $$\begin{array}{c} \xi {\left(f^{-1}\left(\nu \right)\right)}_{x}=\nu_{f(x)}. \end{array}$$

(1) *ξ* is well defined as follows: *f* ^−1^(*ν*)_*x*_ = *f* ^−1^(*v*)_*y*_⇒[*x*]_*f*^−1^(*v*)_*r*__ = [*y*]_*f*^−1^(*ν*)_*r*__⇒*x* + *f* ^−1^(*ν*)_*r*_ = *y* + *f* ^−1^(*ν*)_*r*_⇒*f*(*x* + *f* ^−1^(*ν*)_*r*_) = *f*(*y* + *f* ^−1^(*ν*)_*r*_). Since *f* is a homomorphism, then *f*(*x*) + *f*(*f* ^−1^(*ν*)_*r*_) = *f*(*y*) + *f*(*f* ^−1^(*ν*)_*r*_)⇒*f*(*x*) + *v*_*r*_ = *f*(*y*) + *ν*_*r*_⇒[*f*(*x*)]_*ν*_*r*__ = [*f*(*y*)]_*ν*_*r*__⇒*ν*_*f*(*x*)_ = *ν*_*f*(*y*)_.

(2) *ξ* is a homomorphism:

(25) ξ((f−1(ν))x+(f−1(ν)y))=ξ((f−1(ν))x+y)=νf(x+y)=νf(x)+f(y)=νf(x)+νf(y)=ξ((f−1(ν)))x+ξ((f−1(ν)))y.ξ((f−1(ν))x·(f−1(ν)y))=ξ((f−1(ν))x·y)=νf(x·y)=νf(x)·f(y)=νf(x)·νf(y)=ξ((f−1(ν)))x·ξ((f−1(ν)))y.

(3) *ξ* is an epimorphism; for any *ν*_*y*_ ∈ *S*′/*ν*, since *f* is epimorphism, then there exists *x* ∈ *S*, such that *f*(*x*) = *y*, so *ξ*((*f* ^−1^(*ν*))_*x*_) = *ν*_*f*(*x*)_ = *ν*_*y*_.

(4) *ξ* is monomorphism: *ν*_*f*(*x*)_ = *ν*_*f*(*y*)_⇒[*f*(*x*)]_*ν*_*r*__ = [*f*(*y*)]_*ν*_*r*__⇒*f*(*x*) + *ν*_*r*_ = *f*(*y*) + *ν*_*r*_⇒*f*(*x*) + *f*(*f* ^−1^(*ν*)_*r*_) = *f*(*x*) + *f*(*f* ^−1^(*ν*)_*r*_)⇒*f*(*x* + *f* ^−1^(*ν*)_*r*_) = *f*(*y* + *f* ^−1^(*ν*)_*r*_); since *f* is monomorphism, so *x* + *f* ^−1^(*ν*)_*r*_ = *y* + *f* ^−1^(*ν*)_*r*_; thus [*x*]_*f*^−1^(*ν*)_*r*__ = [*y*]_*f*^−1^(*ν*)_*r*__, so we have (*f* ^−1^(*ν*))_*x*_ = (*f* ^−1^(*ν*))_*y*_; hence *S*/*f* ^−1^(*ν*)≅*S*′/*ν*, and the proof is completed.

## 4. The Relationships between Fuzzy Congruences and Fuzzy Strong *h*-Ideals

In this section, we investigate the relationships between fuzzy congruences and fuzzy strong *h*-ideals of hemirings. The following concepts can be seen in [6].

**Definition 17 .**

A nonempty fuzzy relation *α* on *S* is called a fuzzy equivalence relation if

1. *α*(*x*, *x*) = ⋁_*y*,*z*∈*S*_ *α*(*y*, *z*) (fuzzy reflexive),
2. *α*(*x*, *y*) = *α*(*y*, *x*) (fuzzy symmetric),
3. *α*(*x*, *y*) ≥ ⋁_*z*∈*S*_ *α*(*x*, *z*)∧*α*(*z*, *y*) for all *x*, *y* ∈ *S* (fuzzy transitive).

**Definition 18 .**

A fuzzy equivalence relation *α* on *S* is called a fuzzy congruence if

(26) $$\begin{array}{c} \alpha \left(a+c,b+d\right)\geq \alpha \left(a,b\right)\wedge \alpha \left(c,d\right),\\ \alpha \left(ac,bd\right)\geq \alpha \left(a,b\right)\wedge \alpha \left(c,d\right). \end{array}$$

**Example 19 .**

Consider the set *N*_0_ of all nonnegative integers is a hemiring with respect to the usual addition and multiplication; we define a fuzzy relation on *N*_0_ as follows.

For all *x*, *y* in *N*_0_,

(27) α(x,y) ={1,if  x=y,0.5,if  x≠y  and  both  x,y  are  even  or  both  x,y  are  odd,0,otherwise.

**Theorem 20 .**

Let *α* be a fuzzy congruence on *S* and let *μ*_*α*_ be the fuzzy subset of *S* defined by

(28) $$\begin{array}{c} \mu_{\alpha }\left(x\right)=\underset{x+a+z=y+b+z}{⋁}\alpha \left(a,0\right)\wedge \alpha \left(b,0\right)\wedge \alpha \left(y,0\right). \end{array}$$

If there exist *a*, *b*, *y*, *z* ∈ *S* be such that *x* + *a* + *z* = *y* + *b* + *z*; otherwise, *μ*_*α*_(*x*) = 0. Then *μ*_*α*_ is a fuzzy strong *h*-ideal of *S*.

**Proof**

(1) For any *a*′, *b*′ ∈ *S*, we have

(29) μα(a′+b′) =⋁a′+b′+a+z=y+b+zα(a,0)∧α(b,0)∧α(y,0) ≥⋁a′+c′+z′=e′+d′+z′α(c′,0)∧α(d′,0)∧α(e′,0)  ∧⋁b′+c′′+z′′=e′′+d′′+z′′α(c′′,0)∧α(d′′,0)∧α(e′′,0) =μα(a′)∧μα(b′).

(2) For any *a*′, *b*′ ∈ *S*, we have

(30) μα(b′) =⋁b′+a+z=y+b+zα(a,0)∧α(b,0)∧α(y,0) ≤⋁a′b′+a′a+a′z=a′y+a′b+a′zα(a′a,0)∧α(a′b,0)∧α(a′y,0) ≤⋁a′b′+c′+z′=e′+c′′+z′α(c′,0)∧α(c′′,0)∧α(e′,0) =μα(a′b′).

Similarly, *μ*_*α*_(*a*′*b*′) ≥ *μ*_*α*_(*a*′).

(3) Let *a*′, *b*′, *x*′, *y*′, *z*′ be any elements of *S* such that *x*′ + *a*′ + *z*′ = *y*′ + *b*′ + *z*′; if there exist *c*′, *c*′′, *d*′, *d*′′, *e*′, *e*′′, *z*′′, *z*′′′ ∈ *S* be such that

(31) $$\begin{array}{c} a^{\prime }+c^{\prime }+z^{\prime \prime }=c^{\prime \prime }+d^{\prime \prime }+z^{\prime \prime },\\ b^{\prime }+e^{\prime }+z^{\prime \prime \prime }=e^{\prime \prime }+d^{\prime \prime }+z^{\prime \prime \prime }, \end{array}$$

then we have *x*′ + *c*′′ + *d*′ + *e*′ + *z*′′′ = *y*′ + *c*′ + *e*′′ + *d*′′ + *z*′′′, where *z*′′′′ = *z*′ + *z*′′ + *z*′′′ + *a*′ + *b*′, and so

(32) (y1′+μα)(x′) =⋁x′=y′+cμα(c) =⋁x′=y′+c⋁  c+a+z=y+b+zα(a,0)∧α(b,0)∧α(y,0) ≥α(c′′+d′+e′,0)∧α(e′′+d′′,0)∧α(c′,0) ≥α(c′′,0)∧α(d′,0)∧α(e′,0)∧α(e′′,0)  ∧α(d′′,0)∧α(c′′,0) =α(c′,0)∧α(d′,0)∧α(c′′,0)∧α(e′,0)  ∧α(d′′,0)∧α(e′′,0).

This gives

(33) (y1′+μα)(x′) ≥⋁a′+c′+z′=c′′+d′+z′′α(c′,0)∧α(d′,0)∧α(c′′,0)  ∧⋁b′+e′+z′′=e′′+d′′+z′′α(e′,0)∧α(d′′,0)∧α(e′′,0) =μα(a′)∧μα(b′).

Otherwise, we have *μ*_*α*_(*a*′) = 0 or *μ*_*α*_(*b*′) = 0, and so (*y*_1_′ + *μ*_*α*_)(*x*′) ≥ 0 = *μ*_*α*_(*a*′)∧*μ*_*α*_(*b*′). Summing up the above statements, *μ*_*α*_ is a fuzzy strong *h*-ideal of *S*.

**Remark 21 .**

*μ*_*α*_ is called the fuzzy strong *h*-ideal induced by *α*.

**Theorem 22 .**

Let *μ* be a fuzzy strong *h*-ideal of *S*. Let *α*_*μ*_ be the fuzzy relation on *S* defined by

(34) $$\begin{array}{c} \alpha_{\mu }\left(x,y\right)=\underset{x+a+z=y+b+z}{⋁}\mu \left(a\right)\wedge \mu \left(b\right), \end{array}$$

*x*, *y*, *a*, *b*, *z* ∈ *S*. Then *α*_*μ*_ is a fuzzy equivalence relation on *S*.

**Proof**

Since *μ* is nonempty, it follows that *α*_*μ*_ is also nonempty. Now

(35) αμ(x,x)=⋁x+a+z=x+b+zμ(a)∧μ(b)≥μ(0)∧μ(0)≥μ(u)∧μ(ν),

for any *u*, *v* in *S*. Again *α*_*μ*_(*y*, *z*) = ⋁_*y*+*u*+*z*′=*z*+*v*+*z*′_ *μ*(*u*)∧*μ*(*ν*) for *u*, *v* in *S*. So *α*_*μ*_(*x*, *x*) ≥ *α*_*μ*_(*y*, *z*) for any *y*, *z* ∈ *S*. Hence *α*_*μ*_(*x*, *x*) ≥ ⋁_*y*,*z*∈*S*_ *α*_*μ*_(*y*, *z*); that is *α*_*μ*_(*x*, *x*) = ⋁_*y*,*z*∈*S*_ *α*_*μ*_(*y*, *z*). Therefore, *α*_*μ*_ is fuzzy reflexive. Obviously *α*_*μ*_ is fuzzy symmetric. Now

(36) αμ(x,y)=⋁x+a+z=y+b+zμ(a)∧μ(b)≥⋁x+a+z=x′+a′+z⋁  x′+a′+z=y+b+zμ(a) ∧μ(a′)∧μ(a′)∧μ(b)=⋁x+a+z=x′+a′+z∧μ(a)∧μ(a′) ∧⋁x′+a′+z=y+b+zμ(a′)∧μ(b)=αμ(x,x′)∧αμ(x′,y).

Hence *α*_*μ*_(*x*, *y*) ≥ ⋁_*x*′∈*S*_ *α*_*μ*_(*x*, *x*′)∧*α*_*μ*_(*x*′, *y*). Thus, *α*_*μ*_ is a fuzzy equivalence relation on *S*.

*Open Question*.  Theorem 22 indicates that a fuzzy strong *h*-ideal of *S* can induce a fuzzy equivalence relation on *S*. However, the question whether a fuzzy strong *h*-ideal of *S* can induce a fuzzy congruence on *S* is still open.

## 5. Normal Fuzzy Strong Left *h*-Ideals

In this section, we introduce the normal fuzzy strong left *h*-ideals of hemirings.

**Definition 23 .**

A fuzzy strong left *h*-ideal *μ* of *S* is said to be normal if there exists *x* ∈ *S* such that *μ*(*x*) = 1.

It is obvious to verify that *μ* is normal if and only if *μ*(0) = 1. We also note that any fuzzy strong *h*-ideal containing some normal strong left *h*-ideals is normal. Since if *ν*⊆*μ* and *ν* is normal, then 1 = *ν*(0) ≤ *μ*(0), so *μ*(*x*) = 1.

**Proposition 24 .**

Given a fuzzy strong left *h*-ideal *μ* of *S*, let *μ* ^+^ be a fuzzy set in *S* defined by *μ* ^+^(*x*) = *μ*(*x*) + 1 − *μ*(0) for all *x* ∈ *S*; then *μ* ^+^(*x*) is a normal fuzzy strong left *h*-ideal of *S* which contain *μ*.

**Proof**

Firstly, we prove *μ* ^+^(*x* + *y*) ≥ *μ* ^+^(*x*)∧*μ* ^+^(*y*) for all *x*, *y* ∈ *S*; we have *μ* ^+^(0) = *μ*(0) + 1 − *μ*(0) = 1 and

(37) μ+(x+y)=μ(x+y)+1−μ(0)≥(μ(x)∧μ(y))+1−μ(0)=(μ(x)+1−μ(0))∧(μ(y)+1−μ(0))=μ+(x)∧μ+(y),

which proves (*F*_1_). Then we prove *μ* ^+^(*xy*) ≥ *μ* ^+^(*y*). Similarly,

(38) μ+(xy)=μ(xy)+1−μ(0)≥μ(y)+1−μ(0)=μ+(y).

This proves that (*F*_2_) holds. Hence *μ* ^+^ is a fuzzy left ideal of *S*. Now, let *a*, *b*, *x*, *y*, *z* ∈ *S* be such that *x* + *a* + *z* = *y* + *b* + *z*. Then

(39) (y1+μ+)(x)=⋁x=y+cμ+(c)=⋁x=y+c(μ(c)+1−μ(0))=⋁x=y+cμ(c)+1−μ(0).

Since *μ* is a fuzzy strong left *h*-ideal, we have (*y*_1_ + *μ*)(*x*) = ⋁_*x*=*y*+*c*_ *μ*(*c*) ≥ *μ*(*a*)∧*μ*(*b*), so

(40) ⋁x=y+cμ(c)+1−μ(0) ≥(μ(a)∧μ(b))+1−μ(0) =(μ(a)+1−μ(0))∧(μ(b)+1−μ(0)) =μ+(a)∧μ+(b).

Therefore, *μ* ^+^ is a normal fuzzy strong left *h*-ideal of *S*, and obviously *μ*⊆*μ* ^+^.

**Corollary 25 .**

Let *μ* and *μ* ^+^ be as in Proposition 24. If there exists *x* ∈ *S* such that *μ* ^+^(*x*) = 0, then *μ*(*x*) = 0.

For any strong left *h*-ideal *A* of *S*, the characteristic *χ*_*A*_ is a normal fuzzy strong left *h*-ideal of *S*. It is clear that *μ* is a normal fuzzy strong left *h*-ideal of *S* if and only if *μ* ^+^ = *μ*.

**Proposition 26 .**

If *μ* is a fuzzy strong left *h*-ideal of *S*. Then (*μ* ^+^)^+^ = *μ* ^+^. Moreover, if *μ* is normal, then (*μ* ^+^)^+^ = *μ*.

**Proof**

It is straightforward.

**Theorem 27 .**

Let *μ* be a fuzzy strong left *h*-ideal of *S* and let *f* : [0, *μ*(0)]→[0,1] be an increasing function. Then a fuzzy set *μ*_*f*_ : *S* → [0,1] defined by *μ*_*f*_(*x*) = *f*(*μ*(*x*)) is a fuzzy strong left *h* ideal of *S*. In particular, if *f*(*μ*(0)) = 1, then *μ*_*f*_ is normal; if *f*(*t*) ≥ *t* for all *t* ∈ [0, *μ*(0)], *μ*⊆*μ*_*f*_.

**Proof**

For all *x*, *y* ∈ *S*, then we have

(41) μf(x+y)=f(μ(x+y))≥f(μ(x)∧μ(y))=f(μ(x))∧f(μ(y))=μf(x)∧μf(y),

which proves (*F*_1_). Similarly, *μ*_*f*_(*xy*) = *f*(*μ*(*xy*)) ≥ *f*(*μ*(*y*)) = *μ*_*f*_(*y*), which proves (*F*_2_) holds. Hence *μ*_*f*_ is a fuzzy left ideal of *S*.

Now, let *a*, *b*, *x*, *y*, *z* ∈ *S* be such that *x* + *a* + *z* = *y* + *b* + *z*. Then

(42) (y1+μf)(x)=⋁x=y+dμf(d)=⋁x=y+df(μ(d))=f(⋁x=y+dμ(d)).

Since *μ* is a fuzzy strong left *h*-ideal, we have

(43) (y1+μ)(x)=⋁x=y+dμ(d)≥μ(a)∧μ(b),

so

(44) f(⋁x=y+dμ(d))≥f(μ(a)∧μ(b))=f(μ(a))∧f(μ(b))=μf(a)∧μf(b).

Therefore, *μ*_*f*_ is a fuzzy strong left *h*-ideal of *S*; if *f*(*μ*(0)) = 1, then *μ*_*f*_ is normal; suppose that *f*(*t*) = *f*(*μ*(*x*)) ≥ *μ*(*x*) for all *x* ∈ *S*, which proves *μ*⊆*μ*_*f*_.

Let *N*(*S*) denote the set of all normal fuzzy strong left *h*-ideal of *S*. Note that *N*(*S*) is a poset under the set inclusion.

**Theorem 28 .**

Let *μ* ∈ *N*(*S*) be nonconstant such that it is a maximal element of (*N*(*S*), ⊆). Then *μ* takes only two values 0 and 1.

**Proof**

Since *μ* is normal, we have *μ*(0) = 1. For some *x* ∈ *S*, let *μ*(*x*) ≠ 1; we know that *μ*(*x*) = 0; otherwise, there exists *x*_0_ ∈ *S*, such that 0 < *μ*(*x*_0_) < 1. Now we define on *S* a fuzzy set *ν* by putting *ν*(*x*) = (*μ*(*x*) + *μ*(*x*_0_))/2 for some *x* ∈ *S*. Then *ν* is well defined.

For all *x*, *y* ∈ *S*, we have

(45) ν(x+y)=12(μ(x+y)+μ(x0))≥12(μ(x)∧μ(y))+μ(x0)=12(μ(x)+μ(x0))∧12(μ(y)+μ(x0))=ν(x)∧ν(y),

which proves (*F*_1_) holds.

Similarly, we can obtain

(46) ν(xy)=12(μ(xy)+μ(x0))≥12(μ(y)+μ(x0))=ν(y),

which proves (*F*_2_) holds. Hence *v* is a fuzzy left ideal of *S*. Now, let *a*, *b*, *x*, *y*, *z* ∈ *S* be such that *x* + *a* + *z* = *y* + *b* + *z*; since *μ* is a normal fuzzy strong left *h*-ideal, we have (*y*_1_ + *μ*)(*x*) = ⋁_*x*=*y*+*e*_ *μ*(*e*) ≥ *μ*(*a*)∧*μ*(*b*), which implies

(47) (y1+ν)(x)=⋁x=y+eν(e)=⋁x=y+e12(μ(e)+μ(x0))=12(⋁x=y+eμ(e)+μ(x0))≥12(μ(a)∧μ(b))+μ(x0)=12(μ(a)+μ(x0))∧12(μ(b)+μ(x0))=ν(a)∧ν(b).

Therefore, *ν* is a fuzzy strong left *h*-ideal of *S*. By Proposition 24, *ν* ^+^ is a normal fuzzy strong left *h*-ideal of *S*. Note that

(48) ν+(x0)=ν(x0)+1−ν(0)=12(μ(x0)+μ(x0))+1−12(μ(0)+μ(x0))=12(μ(x0)+1)=ν(0)

and *ν* ^+^(*x*_0_) < 1 = *ν* ^+^(0); this means that *ν* ^+^ is nonconstant and *μ* is not a maximal of *N*(*S*). This is a contradiction.

**Definition 29 .**

A nonconstant fuzzy strong left *h*-ideal *μ* of *S* is called maximal if *μ* ^+^ is a maximal element of *N*(*S*).

**Theorem 30 .**

If a fuzzy strong left *h*-ideal *μ* of *S* is maximal, then

1. *μ* is normal,
2. *μ* takes only the values 0 and 1,
3. *χ*_*μ*^0^_ = *μ*,
4. *μ* ^0^ is a maximal strong left *h*-ideal of *S*.

**Proof**

Let *μ* be a maximal fuzzy strong left *h*-ideal of *S*. Then *μ* ^+^ is a nonconstant maximal element of the poset (*N*(*S*), ⊆). It follows from Theorem 28 that *μ* ^+^ takes only the values 0 and 1. Note that *μ* ^+^(*x*) = 1 if and only if *μ*(*x*) = *μ*(0), and *μ* ^+^(*x*) = 0 if and only if *μ*(*x*) = *μ*(0) − 1. By Corollary 25, we have *μ*(*x*) = 0, and so *μ*(0) = 1. Hence *μ* is normal and *μ* ^+^ = *μ*. This proves that (1) and (2) hold.

(3) Obviously.

(4) It is clear that *μ* ^0^ = {*x* ∈ *S*∣*μ*(*x*) = 1} is a strong left *h*-ideal. Obviously *μ* ^0^ ≠ *S* since *μ* takes two values. Now let *A* be a strong *h*-ideal that contains *μ* ^0^. Then *μ*_*μ*^0^_⊆*μ*_*A*_, and in consequence, *μ* = *μ*_*μ*^0^_⊆*μ*_*A*_. It follows from *μ*being normal that *μ*_*A*_ is also normal and takes only two values: 0 and 1. By the assumption, *μ* is maximal, so *μ* = *μ*_*A*_ or *μ* = *ω*, where *ω*(*x*) = 1 for all *x* ∈ *S*. In the last case *μ* ^0^ = *S*, which is impossible. So, *μ* = *μ*_*A*_; that is, *μ*_*A*_ = *χ*_*A*_. Hence *μ* ^0^ = *A*.

## 6. Conclusion

In this paper, we consider the relationships between fuzzy congruences and fuzzy strong *h*-ideals of hemirings. We also discuss some concept of fuzzy strong *h*-ideals of hemrings and then we consider quotient hemirings and their isomorphism theorem. Finally, we introduce normal fuzzy strong *h*-ideals of hemirings. In the future study of hemirings, we can apply fuzzy congruences of hemirings to some applied fields, such as decision making, data analysis, and forecasting.
